# *Staphylococcus aureus *immunodominant surface antigen B is a cell-surface associated nucleic acid binding protein

**DOI:** 10.1186/1471-2180-9-61

**Published:** 2009-03-26

**Authors:** Nicole M Mackey-Lawrence, Denise E Potter, Nuno Cerca, Kimberly K Jefferson

**Affiliations:** 1 Department of Microbiology and Immunology, Virginia Commonwealth University, Richmond, Virginia, 23298, USA

## Abstract

**Background:**

*Staphylococcus aureus *immunodominant surface antigen B (IsaB) elicits an immune response during septicemia and is generally classified as a virulence factor, but its biological function remains completely undefined. In an attempt to identify staphylococcal RNA-binding proteins, we designed an RNA Affinity Chromatography assay and subsequently isolated IsaB.

**Results:**

Western analysis indicated that IsaB was both secreted and cell-surface associated. Gel Shift analysis confirmed the RNA binding activity but revealed that IsaB bound to any nucleic acid without sequence specificity. IsaB exhibited the highest affinity for double-stranded DNA followed by single-stranded DNA and RNA. Because extracellular DNA has been shown to play a role in biofilm formation, we investigated the biofilm-forming capacity of an isogenic *isaB *deletion mutant but we found that IsaB did not contribute to biofilm formation under any conditions tested.

**Conclusion:**

IsaB is an extracellular nucleic acid binding protein, with little to no sequence specificity, but its role in virulence remains unclear.

## Background

*S. aureus *is one of the leading causes of nosocomial infections and is re-emerging as a major threat among hospitals due to the spread of methicillin resistant strains (MRSA)[1]. Furthermore, the occurrence of community acquired MRSA (CA-MRSA) is on the rise in this country and many others [2]. *S. aureus *has a multitude of virulence factors that allow for host immune evasion, adherence to host tissues, biofilm formation, toxin production, and dissemination during infection [3]. As the biological functions of cellular components continue to be elucidated, [4] more and more virulence factors are added to this extensive list. In a study designed to elucidate potential vaccine targets in *S. aureus*, Lorenz et al identified a protein, which they designated the immunodominant surface antigen B (IsaB), that elicited an immune response during MRSA septicemia. IsaB is a 19.5 kDa *S. aureus *protein with no significant homology to other proteins with known function [5]. Another study demonstrated a mutation in the gene encoding IsaB in a hyper-virulent musculoskeletal isolate, leading the authors to suggest that mutation or loss of IsaB may increase immune evasion in the *S. aureus *isolate under investigation [6]. Other labs have reported microarray data showing that *isaB *expression is increased in response to neutrophil exposure, in biofilms, under anaerobic conditions, and following internalization into human epithelial cells [4,7-9]. All of these phenomena suggest that in spite of its role in eliciting an immune response, IsaB expression is induced during infection. Currently, IsaB is annotated as a putative virulence factor, however its function has yet to be determined.

Biofilms have been shown to be a critical component of certain *S. aureus *infections, as these structures confer increased survival of the bacteria under many stressful conditions such as low nutrient availability, antibiotic challenge, oxidative stress, and host immune defenses [10]. The major intercellular adhesin in *S. aureus *biofilms is the polysaccharide poly-N-acetylglucosamine (PNAG), which is encoded by the intercellular adhesin locus (*ica*) [11,12]. We and others have previously studied the regulation of PNAG production and *ica *expression at the transcriptional level [13-17]. With the aim of characterizing post-transcriptional regulation of *ica *expression, we designed an RNA Affinity Chromatography assay to isolate proteins with high affinity for the 5'-untranslated region of the *ica *transcript. We isolated a single protein, IsaB. Subsequently, we found that IsaB did not play a role in regulation of *ica *expression, was not localized to the cytoplasm where it could potentially play a regulatory role but rather was secreted and partially associated with the bacterial cell surface, and bound to RNA, ssDNA, and dsDNA with no apparent sequence specificity. Because a number of studies have shown a role for extracellular DNA in biofilm formation, we hypothesized that the extracellular DNA-binding protein IsaB could play a role in this process [18-21]. However, we found that IsaB did not contribute to biofilm formation under a variety of conditions. This study is the first to assign a function to the putative virulence factor IsaB. The physiologic role of binding extracellular nucleic acids is still unclear.

## Results

### Isolation of IsaB by RNA Affinity Chromatography

We hypothesized that an RNA-binding protein could regulate *ica *expression at the post-transcriptional level through binding to the 5'-untranslated region (5'-UTR). To isolate factors that bound to the 5'-UTR, we designed an RNA Affinity Chromatography assay using a biotinylated chimeric oligonucleotide (WTUTR-c) based on the sequence upstream from the *ica *locus as shown in Table 1. The 3-nt at the beginning and end were synthesized as deoxyribonucleotides to protect the oligo from exoribonuleases, and the remaining 40-nt were ribonucleotides. The chimeric oligo was immobilized on streptavidin-coated magnetic particles, which were used to isolate proteins from whole cell lysates of *S. aureus *strain MN8. A single 19.5 kDa protein was detectable by Coomassie staining (data not shown), and was identified by Mass Spectral analysis as the immunodominant surface antigen B (IsaB).

**Table 1:** Oligonucleotides used in this study are shown

Oligo name	Sequence
WTUTR-c	5'-BIOTIN-TGCaauuacaaauauuuccguuuaauuauaacaacaaucuauuGCA-3'
IsaBIntein	5'-GGGCATATGAATAAAACCAGTAAAGTTTGTGTAGC-3'
IsaBInteinREV	5'-GGTTGCTCTTCCGCAACCTTTACTTGTTTTGTATGGTGTATGTCC-3'
isaBDELFWD	5'-GGATCCCGGATTTAGGCAATTCTTTTAATGC-3'
isaBDELREV	5'-GGATCCCATTAGAACTAATGTGCTTTGATGG-3'
isaBXhoFWD	5'-GGGCATATGGTTTGTGTAGCAGCAACATTAGC-3'
isaBXhoREV	5'-GGGCTCGAGCGAAGTAACAGTTGGACATACACC-3'
icaUTR6	5'-GUUUAAUUAUAACAACAAUCUAUUGCA-3'
BioticaPRO	5'-BIOTIN-ATTGVGTTATCAATAATCTTA-3'
IcaRcloneFWD	5'-GGTGGGATCCTTGAAGGATAAGATTA-3'
WTUTR(RNA)	5'-Biot-tegugcaauuacaaauauuuccguuuaauuauaacaacaaucuauuGCA-3'

Deoxyribonucleotides are shown in capital letters and ribonucleotides are shown in lower case.

We extensively analyzed the effect of deleting and over-expressing the *isaB *gene on *ica *expression but found that it played no role in this capacity, suggesting that our hypothesis that it was a post-transcriptional regulator of *ica *expression was invalid (data not shown).

### Localization of IsaB

**Figure 1:**
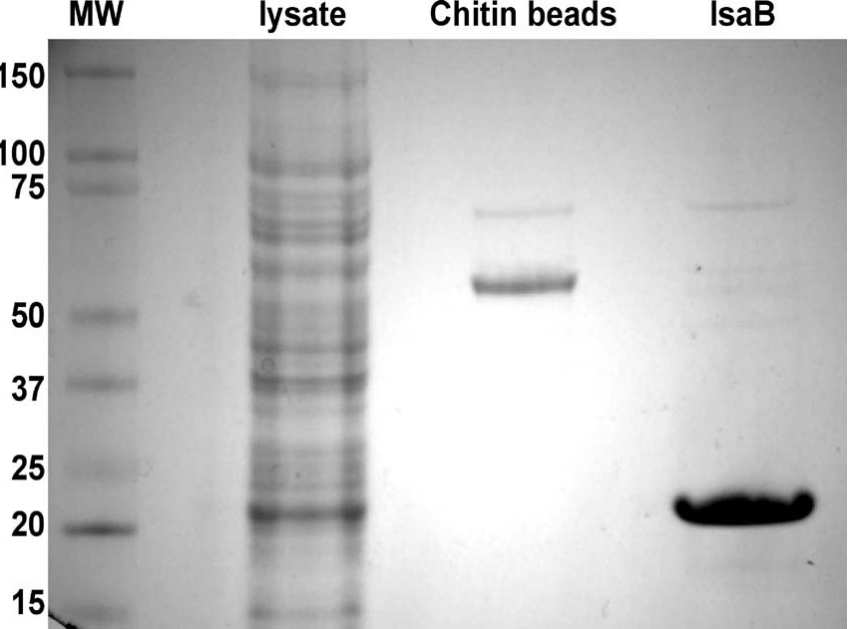
**SDS PAGE analysis of recombinant IsaB**. IsaB-CBD fusion peptide was produced in *E. coli*, purified over a chitin column, and purified, untagged IsaB was cleaved off the column. Lane 1, molecular weight standards; Lane 2, whole cell lysate; Lane 3, CBD tag stripped from chitin beads by boiling in SDS PAGE loading buffer; Lane 4, purified IsaB after CBD cleavage and column elution.

**Figure 2:**
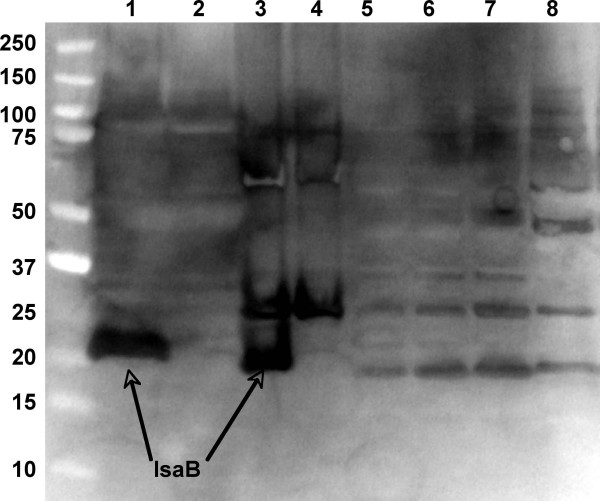
**Cellular localization of IsaB by Western blot analysis**. Sa113 and Sa113Δ*isaB::erm *cultures were fractionated into: spent medium (lanes 1 and 2), cell wall associated (lanes 3 and 4), cell membrane (lanes 5 and 6) and cytoplasmic (lanes 7 and 8) fractions. IsaB bands were observed in both the spent medium and cell wall associated fractions in wild-type Sa113 (lanes 1 and 3, arrows) but not in Sa113Δ*isaB::erm *(lanes 2 and 4 respectively). Proteins that reacted non-specifically with IsaB antiserum were observed in all lanes, but were present in the *isaB *mutant as well as wildtype.

In order to characterize the RNA binding activity of IsaB we cloned the gene into the expression vector pYKB1 and purified untagged protein using a chitin affinity column (Figure 1). Polyclonal antiserum against the purified protein was used to localize IsaB within *S. aureus *(Figure 2). Because the antiserum cross-reacted with other staphylococcal proteins, cellular fractions from an isogenic *isaB *deletion mutant were included for the definitive identification of IsaB bands. IsaB was found in both the spent medium and cell surface extracts of *S. aureus*, while it was absent in both the cell membrane and cytoplasmic fractions.

### Gel shift analysis revealed a lack of sequence specificity by IsaB

**Figure 3:**
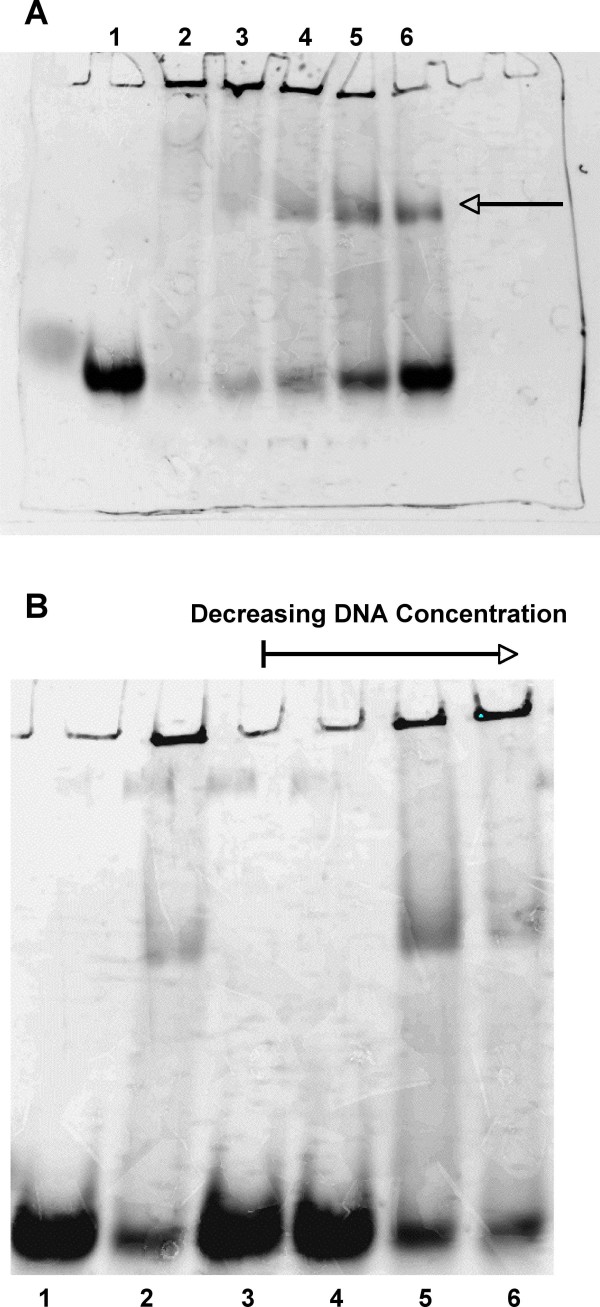
**Electromobility shift analysis of IsaB**. **A**. Purified recombinant IsaB was analyzed by EMSA assay using a fluorescently labeled RNA probe. IsaB shifted the RNA probe in a concentration dependent manner. A. Lane 1, RNA probe alone Lane 2, RNA probe + 3.84 nmol of IsaB, Lane 3, RNA probe + 1.92 nmol of IsaB, Lane 4, RNA probe + 960 pmol of IsaB, Lane 5, RNA probe + 480 pmol of IsaB, Lane 6, RNA probe + 240 pmol of IsaB. At the highest concentrations of IsaB, the RNA probe appeared to aggregate within the wells, while at lower concentrations of IsaB (lanes 4–6) a fraction of the RNA shifted (arrow) but some RNA still remained in the wells. **B**. Effect of salmon sperm DNA on shift; 480 pmol IsaB and 270 pmol labeled. RNA were added to each reaction. Lane 1, RNA probe alone, Lane 2, IsaB, + RNA probe, Lane 3, IsaB + RNA probe and 1.35 nmol unlabeled DNA, Lane 4, IsaB + RNA and 135 pmol unlabeled DNA, Lane 5, IsaB + RNA and 13.5 pmol unlabeled DNA, Lane 6, IsaB + RNA and 1.35 pmol unlabeled DNA.

To confirm the RNA-binding activity of purified IsaB, Electrophoretic Mobility Shift assays (EMSAs) were performed. As shown in Figure 3A, IsaB binds RNA and produces an observable shift. As is commonly noted for nucleic acid binding proteins, in the absence of carrier DNA, much of the probe RNA remained trapped in the well. Addition of sonicated salmon sperm DNA abolished not only retention of the probe within the wells, but the shift as well, indicating that IsaB readily interacted with the carrier DNA. When the ratio of labeled RNA to unlabeled DNA was 2:1, the salmon sperm prevented the shift observed with our labeled RNA oligo (Figure 3B), which suggested a greater affinity of IsaB for the carrier DNA than for the RNA. In order to test the sequence specificity of IsaB, we used a panel of divergent DNA and RNA oligonucleotide probes and found that the nucleic acid-binding activity of IsaB was not specific with regard to sequence (results not shown).

### Gel shift analysis revealed affinity for polymeric RNA and DNA but not nucleotides

**Figure 4:**
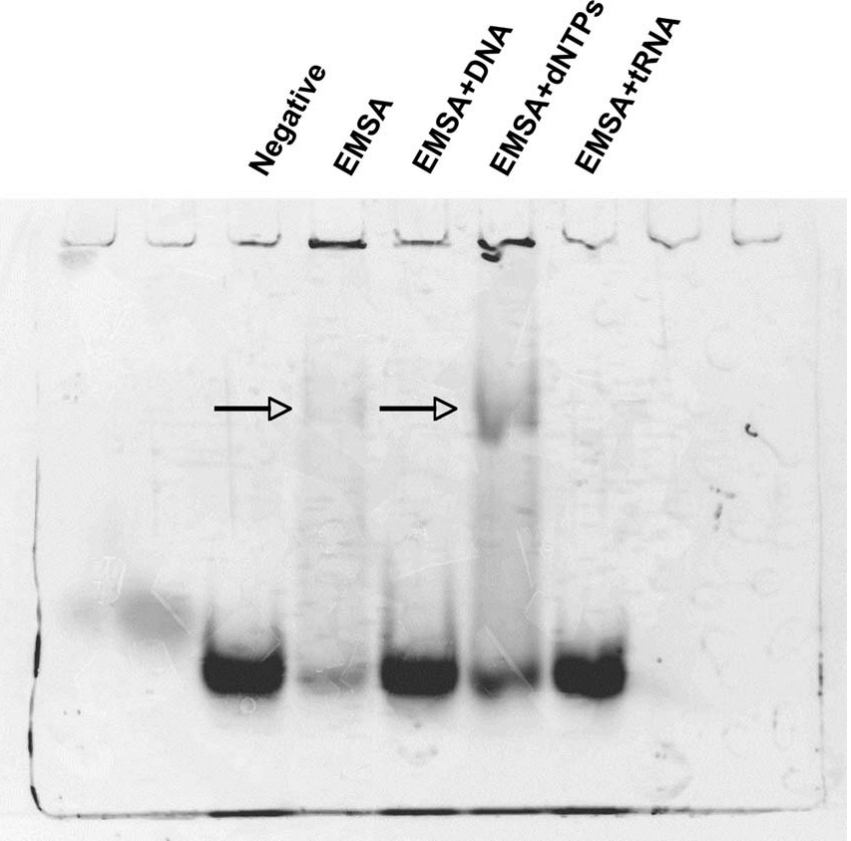
**Competitive Electromobility shift analysis**. EMSAs were performed with unlabeled competitors added to the reactions. 480 pmol IsaB and 270 pmol labeled RNA were included in each sample. Lane 1, labeled probe alone, Lane 2, IsaB + labeled RNA, Lane 3, IsaB + labeled RNA and 270 pmol unlabeled DNA, Lane 4, IsaB + labeled RNA and 270 pmol dNTPs, Lane 5, IsaB + labeled RNA and 270 pmol yeast tRNA.

In order to further characterize the nucleic acid binding activity of IsaB, EMSAs were performed using unlabeled double-stranded DNA (sonicated salmon sperm), yeast tRNA, and deoxyribonucleotides (dNTPs) as competitors (Figure 4). As Figure 4 shows, both yeast tRNA and DNA completely inhibited the IsaB-RNA shift. However, the equivalent concentration of dNTPs was unable to inhibit the shift, indicating that IsaB specifically bound to polymeric nucleic acids and not to free dNTPs.

### BIAcore analysis of IsaB

The affinity of IsaB for nucleic acids was characterized by BIAcore surface plasmon resonance. Using biotinylated DNA, RNA, or double-stranded DNA bait oligonucleotides, we obtained affinities of IsaB to each of these ligands (Table 2). These data, in agreement with the EMSAs, suggest that IsaB binds with the highest affinity to double stranded DNA.

**Table 2:** Dissociation and association constants for binding of IsaB to double-stranded DNA, single-stranded DNA, and RNA as determined by surface plasmon resonance

Ligand	Kd	Ka
Double-stranded DNA	8.10 × 10^-9^	1.23 × 10^8^
Single-stranded DNA	1.08 × 10^-8^	9.28 × 10^7^
RNA	1.65 × 10^-8^	6.07 × 10^7^

### Deletion of *isaB *reduced the accumulation of extracellular DNA on the bacterial cell surface

**Figure 5:**
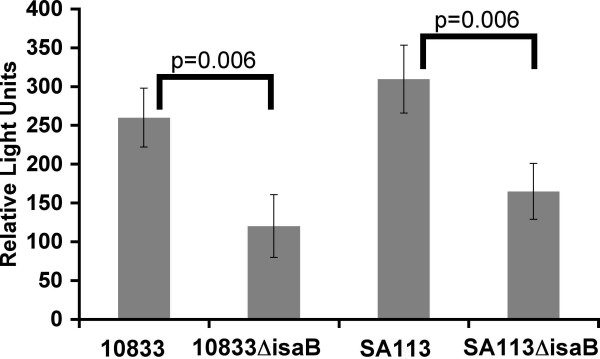
**IsaB binds eDNA on the cell surface**. *S. aureus *strains 10833, Sa113, and their isogenic *isaB *deletion mutants were assayed for their ability to bind to a fluorescently labeled oligonucleotide. The y-axis represents the relative light units. Wildtype fluorescence levels were significantly higher with a probability value of p = 0.006 for 10833 versus 10833Δ*isaB*::ern and Sa113 versus Sa113Δ*isaB*::erm (Student's unpaired T test).

To determine whether native, cell surface-associated IsaB was capable of binding extracellular DNA, wildtype strains 10833 and SA113 and mutants 10833Δ*isaB*::*erm *and SA113Δ*isaB*::*erm *were combined with fluorescently labeled salmon sperm DNA. Relative fluorescence that bound to the bacteria was measured with a fluorimeter. As shown in Figure 5 more fluorescent DNA bound to the wildtype strains. Specifically, there was a 2.2-fold increase in fluorescence of 10833 versus 10833Δ*isaB*::*erm *and a 1.9-fold increase in SA113 versus SA113Δ*isaB*::*erm*.

### Deletion of *isaB *did not affect biofilm formation

**Figure 6:**
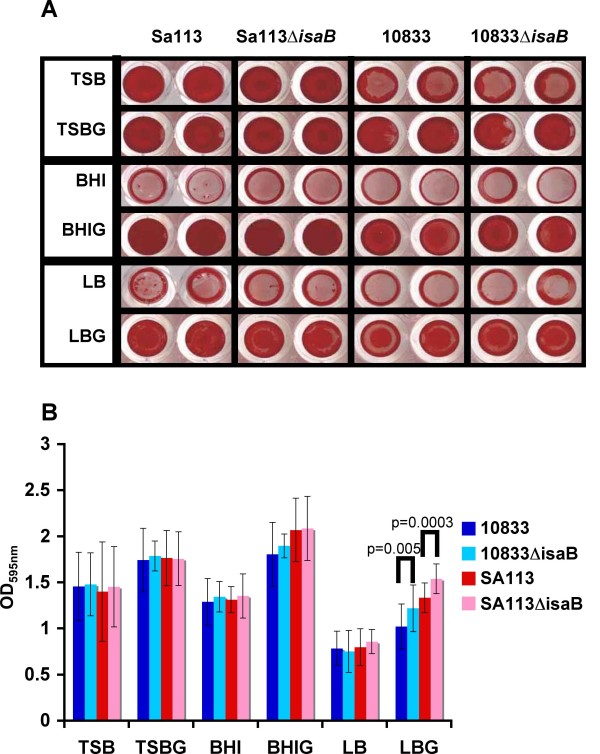
**Microtiter plate assay for biofilm formation**. Strains SA113 and 10833 and their isogenic *isaB *deletion mutants were screened for their ability to form biofilms in different media; TSB, TSB+1% glucose and 3.5% NaCl, BHI, BHI+1% glucose, LB, or LB+1% glucose. **A**. Safranin-stained biofilms and **B**. Average OD_595 nm _values of 8 wells from three separate experiments (24 values) of solubilized safranin-stained biofilms. Deletion of *isaB *did not reduce biofilm formation under any conditions tested but there was a statistically significant increase in OD_595 nm _in the absence of *isaB *in LBG.

Isogenic *isaB *deletion mutants exhibited no apparent growth defects under any conditions tested (data not shown). Microtiter assays for biofilm formation in a variety of media did not reveal any contribution of IsaB to biofilm formation and there was no significant difference between 10833Δ*isaB*::*erm *and SA113Δ*isaB*::*erm *and their respective wildtype parental strains in TSB, TSBG, BHI, BHIG, or LB (Figure 6). Surprisingly, although there was no obvious visible difference, there was a statistically significant increase in the OD_595 nm _in the *isaB *deletion mutants of both strains in LBG. This was consistent between technical and biologic replicates. As extracellular DNA has been shown to affect biofilm development in flow cells [18], we also tested the wildtype and mutant strains under flow conditions. However, there were no observable differences in biofilm formation or maintenance between the *isaB *deletion mutants and their respective wildtype strains (data not shown).

## Discussion

Immunodominant antigen B (IsaB) was first described by Lorenz et al for its immunogenicity in patients recovering from septicemia [5]. While IsaB has been referred to as a virulence factor [7,9], the amino acid sequence does not display significant homology to other proteins of known function, and to date its function remains unknown. In this study we serendipitously discovered the nucleic acid-binding activity of IsaB in a RNA Affinity Chromatography assay designed to identify factors that regulate *ica *expression post-transcriptionally. However, further experiments indicated that while IsaB binds the transcript, it does not affect *ica *expression, and does not play a significant role in the post-transcriptional regulation of *ica*. Furthermore, IsaB was present in the spent medium and associated with the bacterial cell surface but was not found in the cytoplasm where it could function as a post-transcriptional regulatory factor.

To further characterize the RNA-binding activity of IsaB we used EMSAs and found that, while IsaB did bind RNA, the interaction was not sequence-specific and it was also capable of binding to single-stranded and double-stranded DNA. However, we did find that IsaB only binds to polymeric nucleic acids and not to deoxyribonucleotides, suggesting that the nucleic acid binding activity is not a side-effect of a nucleotide-binding site.

IsaB contains an amino-terminal signal peptide and is predicted by PSORTb to be secreted [22]. We found that indeed, IsaB is secreted into the spent medium, but a significant fraction was associated with the cell wall. According to analysis with PSORTb, IsaB lacks an LPXTG motif, so it is not immediately clear how it is retained on the cell surface.

In a recent study Rice et al found that extracellular DNA (eDNA) can contribute to the structural stability of biofilms in *S. aureus*, and that DNase-induced degradation of the eDNA leads to dissolution of the biofilm [18]. Furthermore, IsaB expression was found to be upregulated within biofilms [8], which lead us to hypothesize that binding of eDNA by IsaB could play a role in the establishment or maturation of biofilms, which are a critical component of disease establishment and progression of *S. aureus*. We found, using fluorescently-labeled DNA, that IsaB does play a role in accumulation of extracellular DNA on the bacterial cell surface, however, under our experimental growth conditions, IsaB did not contribute to biofilm-forming capacity. Surprisingly, deletion of *isaB *actually increased biofilm formation slightly, but significantly, in LB containing 1% glucose. This suggests that the role of IsaB may differ depending upon the growth conditions. We are therefore currently exploring the possibility that IsaB may play a more significant role in biofilm formation under more physiologic conditions, and whether or not it contributes to virulence in an animal model of bacteremia.

IsaB elicits an immune response during sepsis, suggesting that it is expressed during infection [5]. Its expression is also induced by neutrophils and following internalization in human epithelial cells, again suggesting expression during infection and a role in virulence [4,7-9]. However, it is not immediately clear how an extracellular DNA-binding protein could play a role in virulence. eDNA present at the site of an infection may come from a variety of sources including lysed neutrophils or neutrophils actively releasing NETs (neutrophil extracellular traps) or from lysed bacterial cells [23,24]. If IsaB does not play a role in biofilm formation, then binding of extracellular DNA to the cell surface could be a mechanism of immune evasion by mimickry or it could result in repulsive forces between the DNA-coated bacteria and the DNA in NETs. We are currently investigating these potential functions of IsaB.

## Conclusion

IsaB is a putative virulence factor and its expression is induced as a result of a variety of conditions related to the *in vivo *disease state. In the data presented here we show that IsaB is an extracellular nucleic acid binding protein with a greater affinity for dsDNA than for ssDNA or RNA. Using isogenic deletion mutants we were unable to demonstrate a role for IsaB on biofilm formation. Further studies are necessary to determine what role IsaB and its nucleic acid-binding activity play in establishment and/or progression of *S. aureus *infection.

## Methods

### Strains and growth conditions

MN8 is a clinical *S. aureus *isolate from a Toxic Shock Syndrome patient, which was isolated by Dr. Patrick Schlievert (University of Minnesota, MN). Strain 10833 is positive for clumping factor (ATCC 25904), is positive for capsular polysaccharide CP5, and is closely related to the sequenced strain Newman. SA113 is closely related to NCTC 8325 and is capsular polysaccharide negative. RN4220 is a restriction deficient laboratory strain from Dr. Richard Novick (Skirball Institute of Molecular Medicine, New York University, NY). The strains were grown at 37°C on tryptic soy agar plates and liquid cultures were either in Luria Bertani broth (LB) or LB+1% glucose (LBG).

### RNA Affinity chromatography

Affinity Chromatography was performed essentially as previously described [13]. *S. aureus *MN8 was grown overnight in 4 L TSB. The bacteria were collected by centrifugation and lysed using a French Pressure cell. A single-stranded chimeric oligonucleotide probe, WTUTR-c was synthesized with a 5' biotin tag; deoxyribonucleotides were included to protect the ends from exoribonucleases (Table 1). 200 nmol of the oligo was immobilized on 10 mg of streptavidin-coated M-280 Dynabeads (Invitrogen, Carlsbad, CA) according to the manufacturer's instructions. The beads were equilibrated with binding buffer-1 (BB-1: 10 mM HEPES, 60 mM KCl, 4 mM MgCl2, 0.1 mM EDTA, 0.1 mg/ml BSA, and 0.25 mM DTT). 1.5 mL lysate (approximately 20 mg of protein) was combined with 6 ml BB1, 0.5 mg sonicated salmon sperm DNA (SSS) and 0.1 mg yeast tRNA, and chilled on ice for 10 min. The lysate mixture was added to the beads and incubated on ice for 10 min. The beads were washed once with BB1+ 0.2 mg/ml SSS, and 10 μg/mL yeast tRNA and twice with BB1 without BSA, SSS, or tRNA. RNA-binding proteins were eluted with 1 ml 10 mM HEPES + 0.25 M KCl. The eluate was concentrated and desalted using Microcon YM-3.5 centrifugal concentrators (Millipore, Billerica, MA). The concentrated sample was subjected to SDS PAGE using NuPAGE 4–15% gradient gels and MOPS buffer (Invitrogen). The gels were stained with Coomassie blue and protein bands were excised and submitted to the Molecular Biology Core Facility (Dana-Farber Cancer Institute, Boston, MA) for sequencing by MALDI-TOF mass spectral analysis.

### Expression of IsaB in *E. coli*

The *isaB *gene excluding the signal sequence was amplified from 10833 total DNA by PCR using primers IsaBIntein and IsaBInteinREV. The PCR product was digested with NdeI and SapI and cloned into the pYKB1 vector (New England Biolabs, Bedford, MA) using Ready-to-go ligase (Amersham, Piscataway, NJ). The pYKB1 vector fuses a carboxy terminal chitin-binding domain (CBD) onto the protein. The ligation reaction was used to transform TOP10 *E. coli *(Invitrogen) and successful transformants were selected by resistance to 50 μg kanamycin/mL. The plasmid was sequenced to confirm the lack of mutations within *isaB *and it was used to transform the BL21-pLysS(DE3)-pRIL strain of *E. coli *(Stratagene, La Jolla, CA). 1 L of LB containing 50 μg kanamycin/mL and 35 μg chloramphenicol/mL was inoculated with 50 mL of overnight culture and incubated at 37°C for 3 hours. The culture was induced with 1 mmol IPTG and incubated 3 hours at 37°C. The bacteria were collected by centrifugation, and resuspended in 25 ml of CBD buffer (20 mM Tris-CL pH 7.0 containing 0.5 M NaCl) with 0.1% Triton X-100 and protease inhibitors (Roche, Indianapolis, IN). The bacteria were lysed using a French pressure cell followed by 6 × 20 sec 9 Watt pulses with a probe-type sonicator. Intact cells and debris were removed by centrifugation, and the supernatant was filtered through a 0.45 μm filter. 8 mL chitin resin (New England Biolabs) was poured into a column, washed once with 10 mL H_2_O and twice with CBD buffer. The lysate was applied to the column, and the column was rinsed 3× with 15 ml of CBD buffer, once with 15 mL CBD buffer containing 1% TritonX-100, 3× with 15 mL CBD buffer, and finally with 15 mL CBD buffer containing 50 mM dithiothreitol (DTT), and the column was incubated 16 hours at 4°C. The column was eluted with 50 mL of CBD buffer and the eluate was concentrated and desalted using 5,000 MW Amicon Ultra concentrators.

Polyclonal antibodies against purified recombinant IsaB were produced in rabbits (Invitrogen) following the company's standard immunization protocol. The polyclonal antiserum was subsequently used for detection of IsaB by western analysis.

### Deletion of *isaB*

We replaced the *isaB *gene with an erythromycin resistance cassette in *S. aureus *strain RN4220 using the pMAD vector (kindly provided by Michel Débarbouillé and Maryvonne Arnaud Pasteur Institute, Paris, France). The *isaB *gene and surrounding sequence were amplified from total DNA from strain 10833 using primers isaBDELFWD and isaBDELREV and the PCR product was cloned into the pCR4-TOPO vector. To delete *isaB*, the plasmid was amplified with primers isaBXhoFWD and isaBXhoREV. The PCR product was treated with DpnI to digest the original methylated plasmid; it was then digested with XhoI and ligated to an erythromycin resistance cassette excised from plasmid pSC57 with XhoI. The region surrounding the *isaB *gene and the intervening *erm *cassette were excised with BamHI and ligated to pMAD. This construct was electroporated into strain RN4220 as described by Lee [25]. Transformants were cultured overnight at 30°C in the presence of 10 μg erythromycin/ml, diluted 1:1000 and sub-cultured overnight at 42°C without antibiotics. The cultures were diluted 1:10, plated on LB agar plates containing 10 μg erythromycin/ml and 200 μg X-gal/ml, and grown for at 42°C. White colonies were picked and screened for the double-crossover event, initially by PCR, and then by DNA sequencing, which was carried out by the Microbiology Core Facility at Harvard Medical School (Boston, MA). The mutation was transduced to strain 10833 using phage 80α [26] to produce strains 10833Δ*isaB::erm *and SA113Δ*isaB::erm*.

### Cellular localization of IsaB

Sa113 and Sa113Δ*isaB*::erm were grown in 1 L TSB for 6–10 hours. Cultures were centrifuged and both the cell pellet and spent medium were collected. Protein from 400 ml spent medium was precipitated by 70% saturation (NH_4_)_2_SO_4_, while stirring at 4°C for 1 hour. Precipitated proteins were collected by centrifugation, the resulting pellet was resuspended in 1 ml of PBS with complete protease inhibitor cocktail tablets (Roche Diagnostics). The samples were dialyzed against 3 L of 0.1× PBS overnight at 4°C before gel electrophoresis.

The cell pellet was washed with PBS and resuspended in 20 ml of Buffer A (40 mM Tris-Cl, 100 mM NaCl, 27% Sucrose, 20 mM MgCl_2_, and protease inhibitor cocktail 1/50 ml). 500 μg lysostaphin was added and the cells were incubated for 4 hours at 37°C. The pellet (protoplasts) and supernatant (peptidoglycan) were separated by centrifugation. The cell pellet was resuspended in 10 ml of water, 1% triton X was added and mixture was rocked for 10 min at RT. Samples were centrifuged 10,000 × g for 20 min to remove intact cells and membranes were collected by centrifugation at 100,000 × g for 1 hr. Following centrifugation the supernatant (cytoplasm) was collected and the pellet (membrane) was resuspended in water. Equal amounts of protein from the four cellular fractions were analyzed by denaturing PAGE using NuPAGE^® ^4–12% Bis-Tris gels (Invitrogen) according to manufacturer's instructions. The proteins were transferred onto a PVDF membrane which was then blocked 1 hr in PBS containing 5% skim milk. The blot was probed with a 1:5,000 dilution of IsaB-specific rabbit antisera in PBS containing 0.05% tween (PBST) and 0.5% skim milk followed by a 1:10,000 fold dilution of goat anti-rabbit horseradish peroxidase conjugated IgG in PBST. Proteins were detected using the ECL Plus detection system (Amersham) and analyzed with a CCD camera (Kodak).

### Electrophoretic mobility shift analysis

Probes for EMSAs were fluorescently labeled with the ULYSIS™ Alexa Fluor^® ^594 Nucleic Acid labeling kit (Invitrogen) according to manufacturer's instructions. Mobility shift reaction mixtures containing 20 μL binding buffer (BB1: 20 mM HEPES, 1 mM DTT, 20 mM KCl, 200 μg BSA/ml, 10% glycerol), 480 pmol purified, recombinant IsaB (optimal concentration determined from Figure 3A, which had either 3.84 nmol, 1.92 nmol, 960 pmol, 480 pmol, or 240 pmol of purified, recombinant IsaB), and 270 pmol RNA probe icaUTR6 were incubated for 10 minutes at room temperature. The reactions were loaded onto a 2% acrylamide gel, bromophenol blue was added to one lane as a marker, and the gel was electrophoresed at 100 V for 30 min. Bands were visualized using a CCD camera. Salmon sperm DNA (SSS) was serially diluted 10-fold and added to designated reactions at final concentrations ranging from 1.35 nmol-1.35 pmol. For inhibition analysis, 2.7 nmol of either salmon sperm DNA (Invitrogen), nucleotides, or yeast tRNA (Sigma, St. Louis, MO) were added in addition to the standard mobility shift reaction mixtures.

### Surface Plasmon Resonance

IsaB interactions with RNA, DNA, and dsDNA were analyzed using a BIAcore Model T100 (BIAcore International, Piscataway, NJ) following manufacturer's instructions. Biotinylated oligos, DNA and RNA, were immobilized on a Streptavidin chip (SA sensor chip, BIAcore International) in 0.33× HBS-EP buffer, supplemented with 1× of non-specific binding inhibitor (BIAcore International). Double-stranded DNA was created by loading the SA DNA coated chip with the complementary strand, icaRcloneFWD. The first flow chamber was left blank to allow for normalization and subtraction of non-specific binding. Resonance units were determined using decreasing concentrations of IsaB that were loaded onto the chip at a flow rate of 30 μl/ml. The kD and kA were determined with the BIA Evaluation Software.

### *S. aureus *binding to fluorescently labeled oligonucleotide

Overnight cultures of *S. aureus *strains 10833 and 10833Δ*isaB::erm *were diluted 1:20 in fresh media (TSB+1% glucose) and incubated at 37°C with shaking. After 4 hours of incubation, approximately 10^8 ^bacteria were collected by centrifugation and resuspended in binding buffer (20 mM HEPES, 1 mM DTT, 20 mM KCl, 200 μg BSA/ml). 40 ng ULYSIS™ Alexa Fluor^® ^488-labeled SSS was added and the reactions were incubated for 15 minutes at room temperature. Control reactions lacked the fluorescent oligonucleotide. Following incubation, the cells were washed once in binding buffer, and resuspended in 200 μl of water. Fluorescent counts were determined using an Flx800 (BioTek, Winooski, VT). Experiments were performed in triplicate and statistical significance was determined using an unpaired T-test.

### Biofilm assays

Biofilm assays were performed essentially as described by Christensen [27]. Overnight cultures of *S. aureus *strains 10833, 10833Δ*isaB::erm*, Sa113, and Sa113Δ*isaB::erm *were diluted 1:20 in fresh media (TSB, TSB+1% glucose +3.5% NaCl, BHI, BHI+1% glucose, LB, or LB+1% glucose) in a microtiter plate. Cultures were incubated overnight at 37°C. The following day, the media was removed, plates were washed with 1× PBS, dried and stained with safranin. Stained biofilms were resuspended in 200 μL water using a probe sonicator and the optical density at 595 nm (OD_595 nm_) was determined using an ELISA plate reader. Values from eight wells per experiment and three separate experiments were averaged. Analysis of biofilm formation over a 48 hr period in flow cells (Stovall, Greensboro, NC) was conducted essentially as described by Rice et al and biofilm thickness was judged visually [18].

## Authors' contributions

NML drafted and wrote the manuscript and performed experiments. DEP performed experiments, NC performed experiments and KKJ conceived of the study and edited the manuscript. All authors have read and approved of the manuscript.

## References

[B1] Gordon RJ Lowy FD Pathogenesis of methicillin-resistant *Staphylococcus aureus *infection Clin Infect Dis 2008 46 Suppl 5 S350 359 18462090 10.1086/533591 PMC2474459

[B2] Voyich JM Otto M Mathema B Braughton KR Whitney AR Welty D Long RD Dorward DW Gardner DJ Lina G Is Panton-Valentine leukocidin the major virulence determinant in community-associated methicillin-resistant *Staphylococcus aureus *disease? J Infect Dis 2006 194 12 1761 1770 17109350 10.1086/509506

[B3] Foster TJ Immune evasion by staphylococci Nat Rev Microbiol 2005 3 12 948 958 16322743 10.1038/nrmicro1289

[B4] Garzoni C Francois P Huyghe A Couzinet S Tapparel C Charbonnier Y Renzoni A Lucchini S Lew DP Vaudaux P A global view of *Staphylococcus aureus *whole genome expression upon internalization in human epithelial cells BMC Genomics 2007 8 171 17570841 10.1186/1471-2164-8-171 PMC1924023

[B5] Lorenz U Ohlsen K Karch H Hecker M Thiede A Hacker J Human antibody response during sepsis against targets expressed by methicillin resistant *Staphylococcus aureus* FEMS Immunol Med Microbiol 2000 29 2 145 153 11024354 10.1111/j.1574-695X.2000.tb01517.x

[B6] Cassat JE Dunman PM McAleese F Murphy E Projan SJ Smeltzer MS Comparative genomics of *Staphylococcus aureus *musculoskeletal isolates J Bacteriol 2005 187 2 576 592 15629929 10.1128/JB.187.2.576-592.2005 PMC543526

[B7] Voyich JM Braughton KR Sturdevant DE Whitney AR Saïd-Salim B Porcella SF Long RD Dorward DW Gardner DJ Kreiswirth BN Insights into mechanisms used by *Staphylococcus aureus *to avoid destruction by human neutrophils J Immunol 2005 175 6 3907 3919 16148137 10.4049/jimmunol.175.6.3907

[B8] Resch A Rosenstein R Nerz C Götz F Differential gene expression profiling of *Staphylococcus aureus *cultivated under biofilm and planktonic conditions Appl Environ Microbiol 2005 71 5 2663 2676 15870358 10.1128/AEM.71.5.2663-2676.2005 PMC1087559

[B9] Fuchs S Pane-Farre J Kohler C Hecker M Engelmann S Anaerobic gene expression in *Staphylococcus aureus* J Bacteriol 2007 189 11 4275 4289 17384184 10.1128/JB.00081-07 PMC1913399

[B10] Jefferson KK What drives bacteria to produce a biofilm? FEMS Microbiol Lett 2004 236 2 163 173 15251193 10.1016/j.femsle.2004.06.005

[B11] Vuong C Kocianova S Voyich JM Yao Y Fischer ER DeLeo FR Otto M A crucial role for exopolysaccharide modification in bacterial biofilm formation, immune evasion, and virulence J Biol Chem 2004 279 52 54881 54886 15501828 10.1074/jbc.M411374200

[B12] Cramton SE Gerke C Schnell NF Nichols WW Götz F The intercellular adhesion (ica) locus is present in Staphylococcus aureus and is required for biofilm formation Infect Immun 1999 67 10 5427 5433 10496925 10.1128/iai.67.10.5427-5433.1999 PMC96900

[B13] Jefferson KK Cramton SE Götz F Pier GB Identification of a 5-nucleotide sequence that controls expression of the ica locus in Staphylococcus aureus and characterization of the DNA-binding properties of IcaR Mol Microbiol 2003 48 4 889 899 12753184 10.1046/j.1365-2958.2003.03482.x

[B14] Cerca N Brooks JL Jefferson KK Regulation of the intercellular adhesin locus regulator (*icaR*) by SarA, {sigma}B, and IcaR in *Staphylococcus aureus* J Bacteriol 2008 190 19 6530 6533 18658265 10.1128/JB.00482-08 PMC2565999

[B15] Maira-Litrán T Kropec A Abeygunawardana C Joyce J Mark G 3rd Goldmann DA Pier GB Immunochemical properties of the staphylococcal poly-N-acetylglucosamine surface polysaccharide Infect Immun 2002 70 8 4433 4440 12117954 10.1128/IAI.70.8.4433-4440.2002 PMC128161

[B16] Conlon KM Humphreys H O'Gara JP *icaR *encodes a transcriptional repressor involved in environmental regulation of *ica *operon expression and biofilm formation in *Staphylococcus epidermidis* J Bacteriol 2002 184 16 4400 4408 12142410 10.1128/JB.184.16.4400-4408.2002 PMC135245

[B17] Dobinsky S Kiel K Rohde H Bartscht K Knobloch JK Horstkotte MA Mack D Glucose-related dissociation between *icaADBC *transcription and biofilm expression by *Staphylococcus epidermidis*: evidence for an additional factor required for polysaccharide intercellular adhesin synthesis J Bacteriol 2003 185 9 2879 2886 12700267 10.1128/JB.185.9.2879-2886.2003 PMC154395

[B18] Rice KC Mann EE Endres JL Weiss EC Cassat JE Smeltzer MS Bayles KW The cidA murein hydrolase regulator contributes to DNA release and biofilm development in *Staphylococcus aureus* Proc Natl Acad Sci USA 2007 104 19 8113 8118 17452642 10.1073/pnas.0610226104 PMC1876580

[B19] Thomas VC Thurlow LR Boyle D Hancock LE Regulation of autolysis-dependent eDNA release by *Enterococcus faecalis *extracellular proteases influences biofilm development J Bacteriol 2008 190 16 5690 5698 18556793 10.1128/JB.00314-08 PMC2519388

[B20] Barken KB Pamp SJ Yang L Gjermansen M Bertrand JJ Klausen M Givskov M Whitchurch CB Engel JN Tolker-Nielsen T Roles of type IV pili, flagellum-mediated motility and extracellular DNA in the formation of mature multicellular structures in *Pseudomonas aeruginosa *biofilms Environ Microbiol 2008 10 9 2331 2343 10.1111/j.1462-2920.2008.01658.x 18485000

[B21] Vlassov VV Laktionov PP Rykova EY Extracellular nucleic acids Bioessays 2007 29 7 654 667 17563084 10.1002/bies.20604

[B22] Gardy JL Laird MR Chen F Rey S Walsh CJ Ester M Brinkman FS PSORTb v.2.0: expanded prediction of bacterial protein subcellular localization and insights gained from comparative proteome analysis Bioinformatics 2005 21 5 617 623 15501914 10.1093/bioinformatics/bti057

[B23] Urban CF Lourido S Zychlinsky A How do microbes evade neutrophil killing? Cell Microbiol 2006 8 11 1687 1696 16939535 10.1111/j.1462-5822.2006.00792.x

[B24] Brinkmann V Reichard U Goosmann C Fauler B Uhlemann Y Weiss DS Weinrauch Y Zychlinsky A Neutrophil extracellular traps kill bacteria Science 2004 303 5663 1532 1535 15001782 10.1126/science.1092385

[B25] Lee JC Electrotransformation of Staphylococci Methods Mol Biol 1995 47 209 216 7550737 10.1385/0-89603-310-4:209

[B26] Kasatiya SS Baldwin JN Nature of the determinant of tetracycline resistance in *Staphylococcus aureus* Can J Microbiol 1967 13 8 1079 1086 6049594 10.1139/m67-144

[B27] Christensen GD Simpson WA Younger JJ Baddour LM Barrett FF Melton DM Beachey EH Adherence of coagulase-negative staphylococci to plastic tissue culture plates: a quantitative model for the adherence of staphylococci to medical devices J Clin Microbiol 1985 22 6 996 1006 3905855 10.1128/jcm.22.6.996-1006.1985 PMC271866

